# Heritability of Horn Size in Thinhorn Sheep

**DOI:** 10.3389/fgene.2019.00959

**Published:** 2019-10-11

**Authors:** Zijian Sim, David W. Coltman

**Affiliations:** ^1^Department of Biological Sciences, University of Alberta, Edmonton, AB, Canada; ^2^Fish and Wildlife Forensic Unit, Alberta Fish and Wildlife Enforcement Branch, Government of Alberta, Edmonton, AB, Canada

**Keywords:** fitness-related trait, heritability, Genome-Wide Association Study, horn size, thinhorn sheep

## Abstract

Understanding the genetic basis of fitness-related trait variation has long been of great interest to evolutionary biologists. Secondary sexual characteristics, such as horns in bovids, are particularly intriguing since they can be potentially affected by both natural and sexual selection. Until recently, however, the study of fitness-related quantitative trait variation in wild species has been hampered by a lack of genomic resources, pedigree, and/or phenotype data. Recent innovations in genomic technologies have enabled wildlife researchers to perform marker-based relatedness estimation and acquire adequate loci density, enabling both the “top-down” approach of quantitative genetics and the “bottom-up” approach of association studies to describe the genetic basis of fitness-related traits. Here we combine a cross species application of the OvineHD BeadChip and horn measurements (horn length, base circumference, and volume) from harvested thinhorn sheep to examine the heritability and to perform a genome-wide single-nucleotide polymorphism association study of horn size in the species. Thinhorn sheep are mountain ungulates that reside in the mountainous regions of northwestern North America. Thinhorn sheep males grow massive horns that determine the social rank and mating success. We found horn length, base circumference, and volume to be moderately heritable and two loci to be suggestively associated with horn length.

## Introduction

The genetic basis of trait diversity is a fundamental area of inquiry in evolutionary biology. Knowledge regarding the generation, inheritance, and maintenance of variation strikes at the core of our understanding of evolution. Genes that underlie fitness-related traits are of particular interest since selection is thought to act most strongly on these relationships (Ellegren and Sheldon, 2008). Key areas of inquiry include questions on the role of additive genetic variation (Lynch and Walsh, 1998) and the elucidation of genomic architectures (Slate et al., 2009; Slate et al., 2010) in quantitative traits. Until recently, the study of fitness-related quantitative trait variation has been hampered by a lack of genomic resources, pedigree, and/or phenotype data and has thus been restricted to either the laboratory or a select few wild species under long-term study (Kruuk and Hill, 2008; Slate et al., 2010).

Recent advances in genomic technologies have ushered in a new age of inquiry into the genetic basis of traits by dramatically lowering the per-unit cost of obtaining genetic data, particularly for nonmodel organisms (Davey et al., 2011; Helyar et al., 2011). This drop in price and ease of genetic data collection have advanced our ability to feasibly investigate a large-enough number of loci to reasonably interrogate the genome for associations with phenotype (Garvin et al., 2010). Furthermore, new methods in relatedness estimation enabled by large genomic datasets have also allowed us to overcome the imprecision of those estimated using smaller marker sets and thus the need for difficult to obtain pedigree information (Coltman, 2005; Csilléry et al., 2006; Gienapp et al., 2017). These key gains in our ability to perform marker-based relatedness estimation and acquire adequate loci density enabled by second- and third-generation DNA technologies open the door for us to utilize both the “top-down” approach of quantitative genetics (Gienapp et al., 2017) and the “bottom-up” approach of association studies (Santure and Garant, 2018) to describe the genetic basis of fitness-related traits.

Secondary sexual characteristics are intriguing targets of inquiry since they are potentially under both natural and sexual selection. While beneficial alleles may be expected to be driven to fixation by either process, the presence of both may yield counterbalancing selection pressures that maintain variation (Kruuk et al., 2008). Horns in bovids, such as thinhorn sheep, are striking examples of a secondary sexual characteristic subject to sexual selection. Male mountain sheep grow massive horns that make up 8% to 12% of their body weight (Feldhamer et al., 2003). During the mating season, large horned males are more dominant and more likely to mate (Geist, 1971; Hogg, 1984; Coltman et al., 2002). The same relationship is not observed in females (Favre et al., 2008). From a management perspective, horn length (along with age) is one of two components that determine the legal status of a ram for harvest in most jurisdictions.

Quantitative genetic studies of the closely related bighorn sheep have found horn size to be moderately to highly heritable (Coltman et al., 2003; Coltman et al., 2007; Poissant et al., 2008; Miller et al., 2018). Microsatellite-based QTL mapping has also found regions suggestively associated with horn morphology (Poissant et al., 2011). A region identified as a possible QTL for horn dimension in Poissant et al. (2011) was also found by a genome resequencing study to show signatures of a selective sweep in a separate population of bighorn sheep (Kardos et al., 2015). This region contains the gene coding for Relaxin-like receptor 2 (*RXFP2*), which has been shown to strongly influence horn development in domestic sheep and underwent strong positive selection due to artificial breeding for individuals lacking horns (Kijas et al.). In a feral breed of domestic sheep, the Soay sheep of St. Kilda archipelago (Scotland), *RXFP2* has been found to be strongly associated with discrete and quantitative variation in horn phenotype (Johnston et al., 2011). A more recent study found that variation in *RXFP2* in Soay sheep is maintained by a life history trade-off —allele conferring greater fecundity is associated with lower survival (Johnston et al., 2013). *RXFP2* has been found in humans and mice to be positively correlated with testosterone levels in blood, while mutations in *RXFP2* have been found to be associated with osteoporosis (Ferlin et al., 2008) and testicular descent (Feng et al., 2009) in mice and humans.

To date, no study has investigated the heritability or additive genetic variance of a fitness-related trait in thinhorn sheep. While some studies have sought to investigate candidate loci for association with pelage color (Loehr et al., 2008) and signatures of selection (Worley et al., 2006), none has yet performed a genome-wide association study of a sexual selected trait with thousands of single-nucleotide polymorphisms (SNPs). In this study, we combine a cross-species application of a high-density domestic SNP array, the OvineHD BeadChip, and horn measurements collected during regulatory inspections of harvested thinhorn sheep to examine the genetic architecture of horn size in thinhorn sheep. First, we estimate the heritability of three horn size metrics: (1) horn length, (2) base circumference, and (3) volume using an “animal model” (Henderson, 1984; Kruuk, 2004), a linear mixed-effects model first used in animal breeding. Then, we perform a genome-wide association analysis between SNP markers and each of the three horn size metrics. This is first study of its kind for thinhorn sheep and one of the few for a wild species not under long-term study.

## Methods

### Sample Origins and Horn Measurements

**Figure 1:**
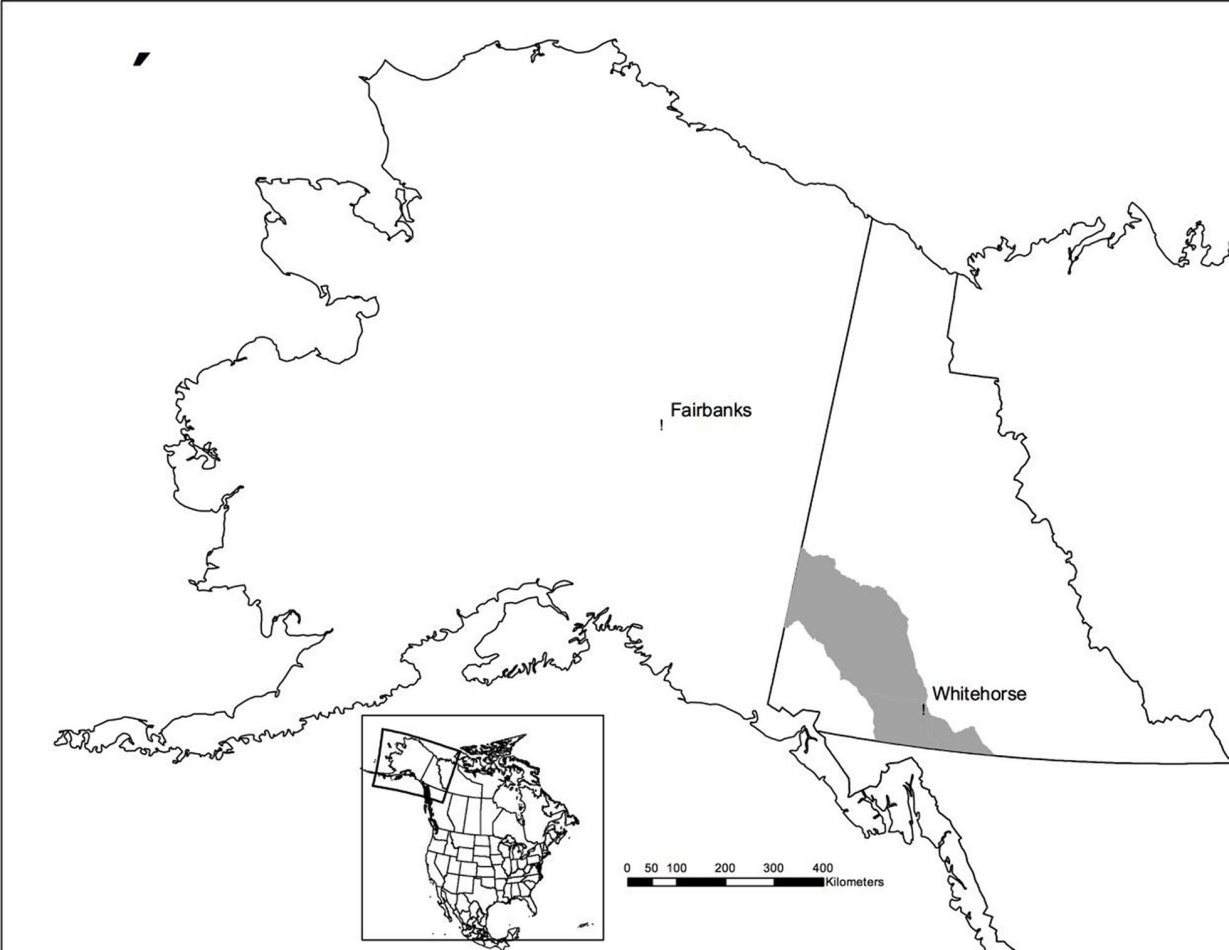
Map of study area. Shaded region represents region in southwest Yukon where the samples originated.

Horn measurements and tissue samples from 192 individuals were collected from 2013 to 2015 from hunter-harvested Dall’s sheep (*Ovis dalli dalli*) in game management units 5, 7, and 9 in Yukon, Canada (**Figure 1**), submitted as part of regulatory compliance to jurisdictional authorities. Dall’s sheep are a northern subspecies of thinhorn sheep, which is one of two closely related mountain sheep species in North America (Valdez and Krausman, 1999). Dall’s sheep are notable in being the only white-colored mountain sheep subspecies in North America. Populations of Dall’s sheep occupy the mountainous regions in Alaska, Yukon, western Northwest Territories, and northwestern British Columbia (Feldhamer et al., 2003).

**Figure 2:**
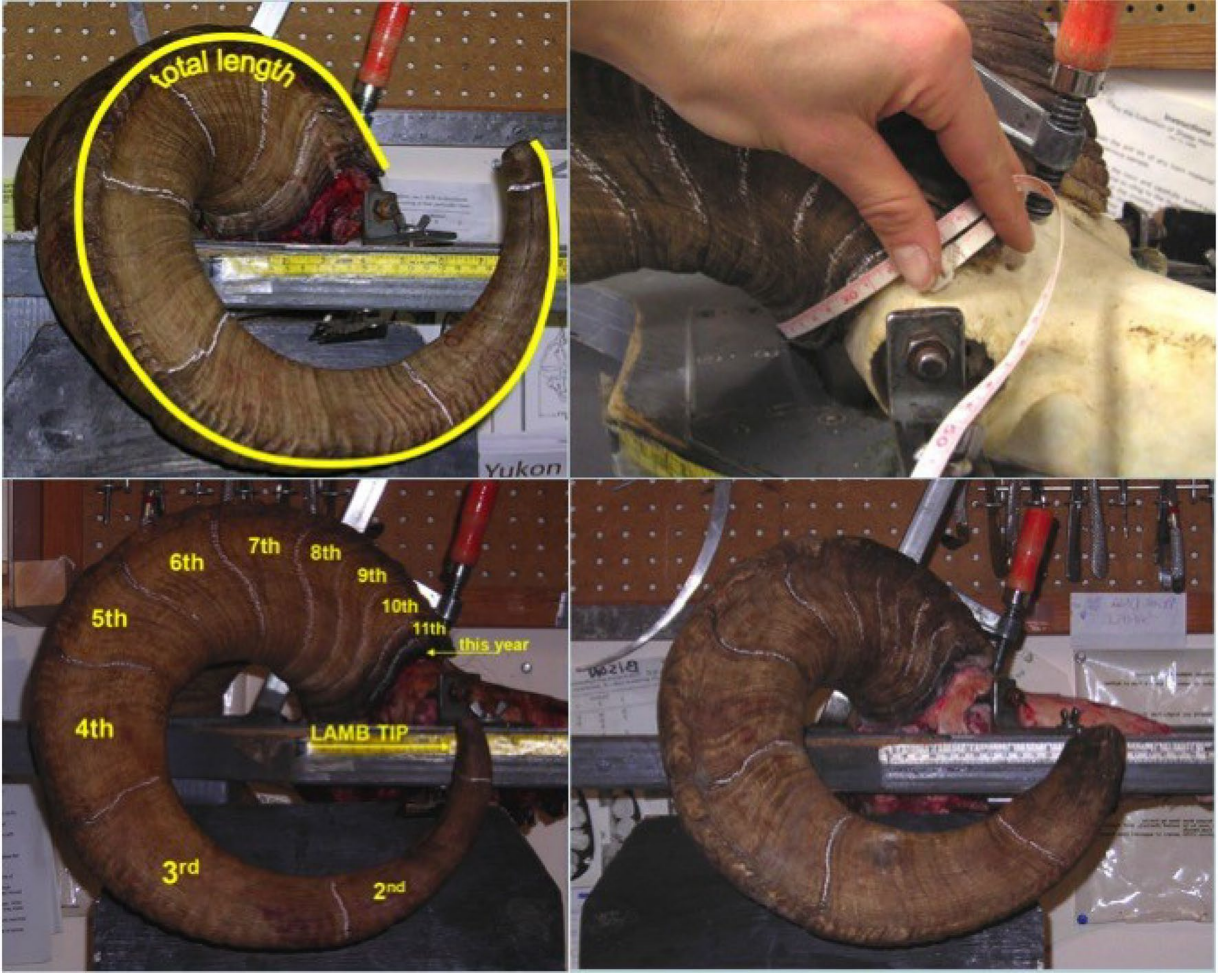
Pictures showing the horn measurement process: **(A)** Total horn length measurement, **(B)** horn base circumference, **(C)** marking of annuli to determine age, and **(D)** example of horn with broken/worn lamb tip. Note that horns in **(A)**, **(B)**, and **(C)** are considered full-curl. (Image courtesy of Yukon Ministry of Environment.)

Hunting regulations in Yukon stipulate that only rams over the age of 8 or possessing horns that “extend beyond a line running from the center of the nostril to the lowermost edge of the eye” (also known as full curl) may be legally harvested (Yukon Hunting Regulations 2018–2019; **Figure 2**). Hunters are required to submit harvested Dall’s sheep rams for registration during which measurements for (1) horn length, (2) horn base circumference, and (3) annuli length are taken. Horn length is measured from tip to base of the horn following the outside curvature of the horn using a flexible measuring tape. The longer of the right and left horn is reported. Horn base circumference is the circumference measurement of each annual growth segment. Horn growth in Dall’s sheep occurs in between April to September according to the seasonal patterns of North America (Bunnell, 1978). The cessation of horn growth after the growing season creates annual growth rings, or annuli, which can be used to estimate the age of an individual (Geist, 1966; Hemming, 1969). Annuli length is the measurement between two growth segments. Annuli can be used to estimate the horn dimensions at the end of each preceding growing season using the proxy of annuli as representing the horn base for those years. We estimated the horn volume using the formula for a conical frustum: volume = 1/3π*H*(*r*1^2 + *r*1*r*2 + *r*2^2), where *r*1 and *r*2 represent the base radii at either end of an annual growth segment, and *H* is the length of the segment (Heimer and Smith Iii, 1975).

### Quality Control

The horns of most rams show some degree of wear or less commonly breakage, which may make the first annulus difficult to distinguish. A missed first annulus will lead to the growth of year 1 (lamb tips) and year 2 growth to be recorded as a single growth increment, resulting in an overestimate of year 1 growth and underestimate for the subsequent years. Furthermore, age determination will also be in error. To mitigate the effect of misidentified or missing first annulus, we removed individuals with biologically implausible growth increments, that is, year 1 growth >160 mm and sum of year 1 and 2 > 420 mm (Bunnell, 1978; Hik and Carey, 2000). Horns with a visible first annulus but worn lamb tips may still result in an underestimate of year 1 growth. Therefore, we also (1) removed all measures of year 1 horn length and base circumference, (2) subtracted year 1 growth (tip to first annulus) for all horn length measurements, and (3) did not calculate the horn volume of the lamb tips.

### SNP Genotyping and Quality Control

We extracted DNA from 192 samples of Dall’s sheep rams with the Qiagen DNeasy Blood and Tissue Kit (Qiagen) using standard protocol. We quantified the extracted DNA using the Qubit Fluorometer (Life Technologies) and normalized to 50 ng/µl in preparation for genotyping. We genotyped the sampled individuals using the Ovine Infinium^®^ HD SNP BeadChip following the manufacturer’s protocol (Qiagen). The Ovine Infinium^®^ HD SNP BeadChip is an SNP array containing 606,006 markers originally designed for use in domestic sheep (*Ovis aries*) by the International Sheep Genomics Consortium (ISGC) (Kijas et al., 2014). *O. aries* and *O. dalli* are thought to have diverged ∼3.1 million years ago (Bunch et al., 2006). Raw signals were converted into genotype calls using a custom cluster file provided by the ISGC, which was developed using a multibreed panel of 288 *O. aries* individuals (J. McEwan, unpublished), using the software GENOMESTUDIO (Illumina). We also used GENOMESTUDIO to cull low-quality genotype calls using a GenCall (GC) score threshold of 0.8. The GC score of genotype call is an assessment of cluster quality based on how tightly clustered the raw signal of that genotype call is compared to other calls of identical genotype and can range from 0 to 1 (higher is better). Post GC score quality-controlled genotype calls were exported in PLINK format using a custom plug-in (Illumina).

We used PLINK v1.07 (Purcell et al., 2007) to remove all individuals or loci with genotyping rate of <0.9, x-linked loci (based on assumed synteny with domestic sheep), and/or minor allele frequency of <0.01. We also performed a check for Hardy–Weinberg equilibrium (HWE; α = 0.001) but did not remove any loci due to HWE deviations since loci under selection, thus potentially associated with horn size, are expected to be out of HWE (Turner et al., 2011). We visually inspected the clustering quality for loci significantly associated with horn size by viewing the SNP graphs for those loci in GENOMESTUDIO (**Figure S1**).

### SNP-Based Quantitative Genetics and Genome-Wide Association

We used the R packages GENABEL v1.8 (Aulchenko et al., 2007; Karssen et al., 2016) and its extension for repeated measures, REPEATABEL v1.1 (Rönnegård et al., 2016), to perform a genome-wide association study and estimate variance components for phenotypic variation.

Repeated-measures data generally result from longitudinal studies; however, annualized pattern growth due to the cessation of horn growth each winter allows for the estimation of horn measurements in previous years. The use of repeated measures allows for better estimates of within-individual variation and has been found to increase the power of genome-wide association studies by providing year-to-year variation in a trait measurement (Rönnegård et al., 2016). The method implemented in REPEATABEL first fits a linear mixed model (with no SNP effects) that includes additive genetic (*V*_a_), permanent environment (*V*_pe_), cohort (*V*_yb_), year of measurement (*V*_ym_), and residual variation (*V*_r_) as random effects and age associated with each horn size measurement as a fixed effect. Horn size variation is thus broken down into five components, *V*_p_ = *V*_a_ + *V*_pe_ + *V*_yb_ + *V*_ym_ + *V*_r_. Permanent environmental effect was calculated from repeated measures of the same individual to account for variation associated with environment condition effects specific to that individual. Additive genetic variation was estimated using a genomic relationship matrix (GRM) constructed by calculating the proportion of shared alleles at each locus weighted by allele frequencies. Narrow sense heritability was calculated as the ratio of additive genetic variation and overall phenotypic variation (*h* ^2^ = *V*_a_/*V*_p_). Next, the covariance structure of our linear model is used to test for associations of individual SNPs with horn size measures using ordinary least squares using REPEATABEL. *P* values for SNP associations were calculated using Wald tests. We defined genome-wide significance and suggestive significance of SNP association using 0.05/nSNPs and 1/nSNPs, respectively (nSNPs − number of markers). In association analysis containing repeated measures of potentially related individuals, there are concerns that significance may be inflated because (1) population stratification may overestimate SNP effects and (2) repeated measures of the same individual may be correlated. By using the (co)variance matrix constructed in the first model-fitting step, we can account for relatedness by using the GRM and within individual variance *via* the estimation of permanent environmental effects (Rönnegård et al., 2016). We calculated the genomic inflation factor (λ) (post genomic control) for each model by using GENABEL to perform a regression analysis of observed versus expected *P* values (Aulchenko et al., 2007). We reviewed gene annotations of significantly associated loci in the *O. aries* genome (assembly 3.1, ISGC).

## Results

### Data Acquisition and Quality Control

**Figure 3:**
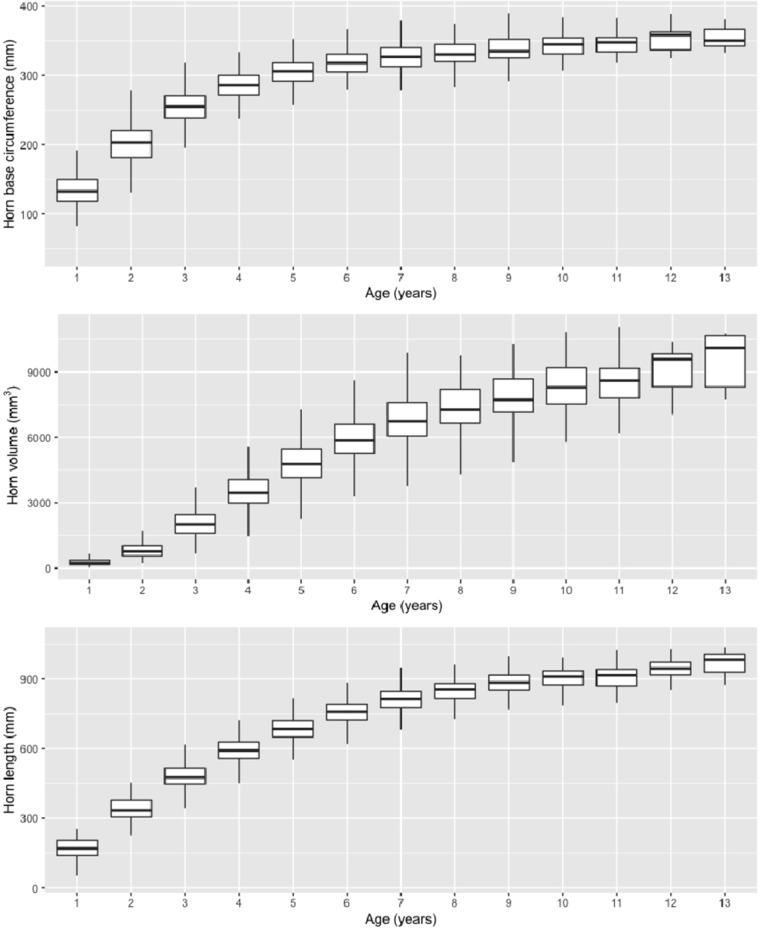
Plots of horn size traits as function of age class.

We acquired SNP genotypes and horn size measurement data for 192 Dall’s sheep rams from southwestern Yukon, Canada (**Figure 3**, **Table 1**). Five individuals were excluded because of low call rates (<0.9) resulting in an overall per-individual call rate of >0.989 for the remaining individuals. Subsequently, we excluded 131,836 loci due to poor cluster quality (GC score <0.8), 6,580 due to low locus-specific call rate (call rate <0.9), 154 for being x-linked, and 460,801 for having minor allele frequency (MAF) < 0.01, resulting in an SNP dataset of 6,635 loci representing each chromosome. This degree of polymorphism is in line with other wild sheep studies employing domestic sheep based SNP chips for genotyping (Miller et al., 2011; Sim et al., 2016). A further seven individuals were culled for biologically implausible and/or potential recording errors resulting in a final dataset of 180 individuals genotyped at 6,635 SNPs.

**Table 1:** Number of individuals (*N*_ind_), number of observations (*N*_obs_), means, variance, and estimated random effect sizes of three horn size traits in Dall’s sheep.

Trait	*N*_ind_	*N*_obs_	Mean (SD)	*V*_obs_	V_p_(SE)	*h* ^2^ (SE)	*V*_pe_ (SE)	*V*_yrmeas_ (SE)	*V*_yrbirth_ (SE)	*V*_r_ (SE)
Horn length	180	1,647	627.7 (243.0)	59,032	2,926(52.7)	0.33 (0.02)	0.35 (0.02)	0.02 (0.01)	0.01 (0.01)	0.29 (0.03)
Horn base circumference	180	1,695	280.1 (69.5)	4,833	542(14.1)	0.36 (0.03)	0.29 (0.03)	0.06 (0.02)	0.01 (0.01)	0.28 (0.04)
Horn volume	177	1,616	4,525.9 (2,953.9)	8,725,770	796,253 (14,332.6)	0.36 (0.02)	0.31 (0.02)	0.01 (0.01)	0.01 (0.01)	0.34 (0.03)

V_Obs_ represents the observed variance in the original data, while V_P_ is the phenotypic variance, which is obtained by summing the variance components estimated for each model.

### SNP-Based Quantitative Genetics and GWAS

**Figure 4:**
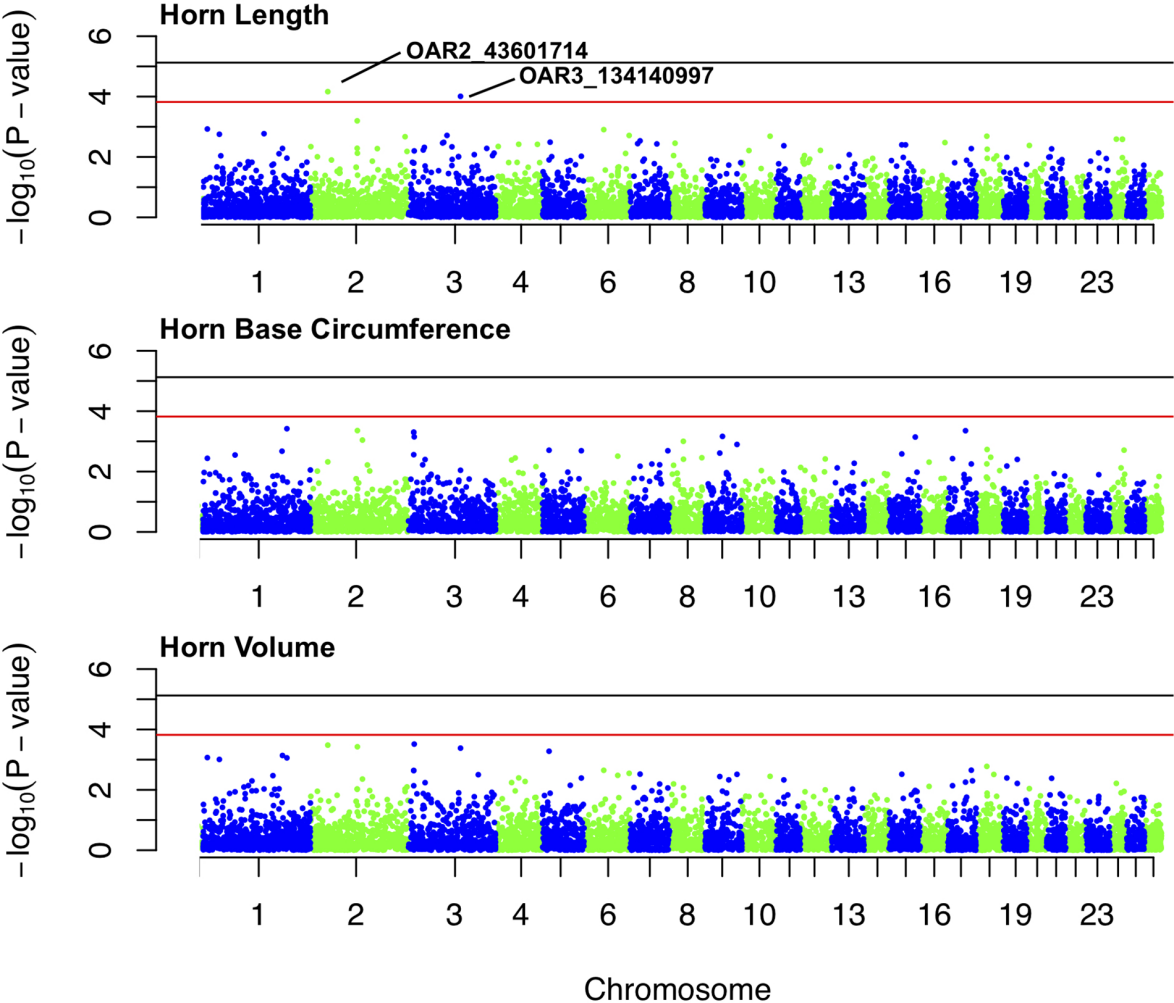
Manhattan plots for associations between SNPs and each of three horn size traits: horn length, horn base circumference, and horn volume. The black line represents the threshold for genome-wide significance, and red line represents suggestive significance. The two labeled loci are of suggestive significance for horn length.

**Figure 5:**
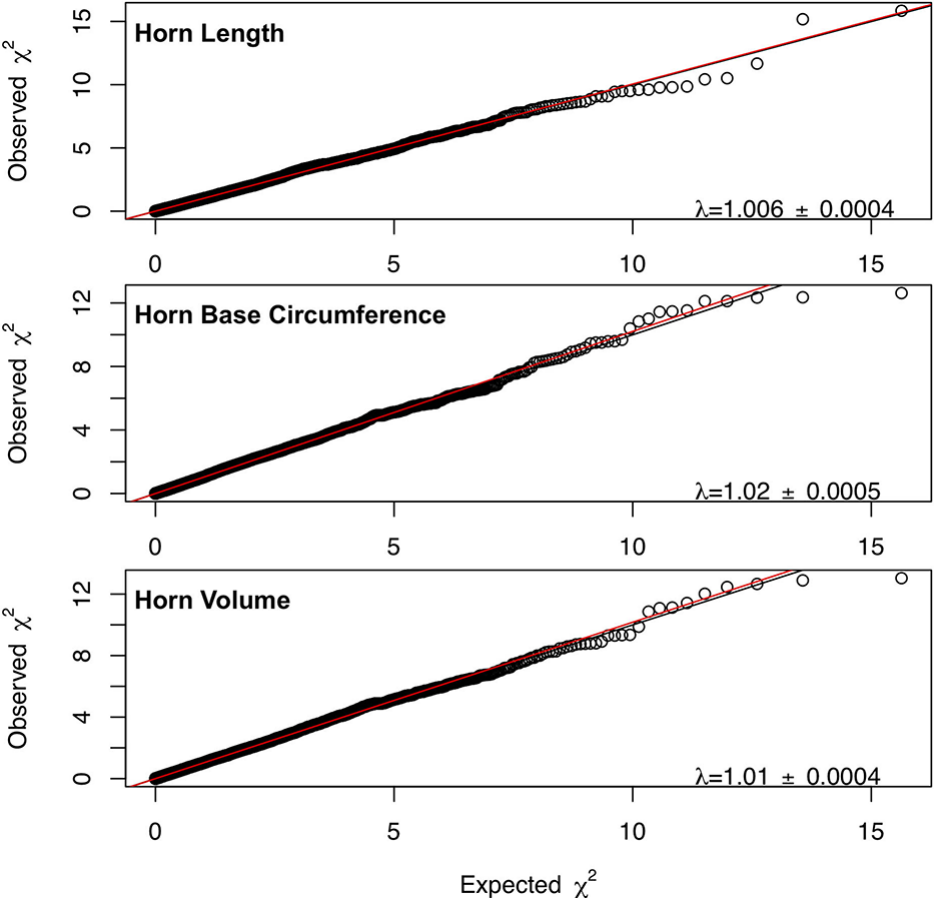
Q-Q plots for each horn size trait with the genome inflation factor and standard error printed on the bottom right corner of each plot. The black line represents a 1:1 correspondence, while the red line is a regression through the observed data.

Marker-based estimates of heritability for horn measures ranged from 0.33 to 0.36 (**Table 1**). Of the nongenetic effects, we found small but significant effects for year of birth and year of measurement (0.01–0.06), while permanent environmental effects were relatively larger (0.29–0.31). Manhattan plots for traits measured and associated Q-Q plots are shown in **Figures 4** and **5**, respectively. We found little to no evidence of genomic inflation post genomic control (all λ ∼1; **Figure 5**).

No loci were found to be associated with any of the horn size traits at the genome-wide significance level (**Table S1**-**S3**). Two loci, (1) OAR2_43601714 and (2) OAR3_134140997 (both in HWE), showed suggestive association with horn length (**Figure 4**). The minor allele frequencies of the two loci are 0.03 and 0.04, respectively. OAR2_43601714 is located on chromosome 2 in the gene *GFRA2* (GDNF family receptor α). In domestic sheep, *GFRA2* codes for receptors that interact with glial cell line–derived neurotrophic factors (GDNFs) (Jing et al., 1997). OAR3_134140997 is located on chromosome 3 in the putative gene *FIGNL2* (fidgetin-like protein 2). In mammals, fidgetin-like proteins belong to a family of ATPases associated with embryonic development (Cox et al., 2000; Frickey and Lupas, 2004).

## Discussion

In this study, we investigated the genetic basis of fitness-related traits in Dall’s sheep by genotyping 192 animals using a cross-species application of a domestic sheep high-density SNP array (>600,000 loci). We achieve a typical conversion rate of about 1% (Miller et al., 2012; Sim et al., 2016), which resulted in an SNP panel of ∼6,000 loci, comparable to that of a medium-density SNP array. By combining the genotype data with horn dimension measurements taken during compulsory inspections of harvested rams, we show that horn length, base circumference, and volume are moderately heritable in thinhorn sheep. Point estimates of narrow sense heritability ranged from 0.33 to 0.36. This level of heritability is comparable to estimates of similar traits using pedigree data in the closely related bighorn sheep (*Ovis canadensis*). For example, Miller et al. (2018) found horn length and base circumference to be moderately heritable (0.15 and 0.23, respectively) in a population of bighorn in Ram Mountain, Alberta, Canada, while Poissant et al. (2008) found heritability of male horn volume to be 0.32 in the same bighorn sheep population. Studies of quantitative traits using genome-wide SNP data in place of pedigrees in other wild mammalian (Malenfant et al., 2018) and avian (Kardos et al., 2016; Lundregan et al., 2018) species have also yielded similar heritability values.

We found two loci of suggestive association with horn length that mapped to genes *GFRA2* and *FIGNL2* in the domestic sheep genome. In humans, *GFRA2* is a protein-coding gene for a coreceptor in the *GDNF* family of neurotrophic ligands (Jing et al., 1997). This family of ligands is involved in transmembrane signal transduction and plays an important role in the development of the central and peripheral nervous system (Lin et al., 1993). *FIGNL2* is less well studied and a putative gene inferred from the human genome. In humans, *FIGNL2* is thought to be a paralog of the gene fidgetin (*FIGN*), which belongs to a superfamily of genes that code for ATPases associated with diverse cellular activities proteins (AAA proteins). This superfamily is made up of a wide range of molecular chaperones that facilitate cellular functions such as proteolysis and membrane fusion characterized by a common conserved ATP-binding domain of ∼240 residues (Lupas and Martin, 2002).

While biologically plausible *post hoc* rationales can be invoked regarding the mechanism underlying these associations, we do not see an immediate connection. This, the suggestive nature of the statistical association, and the relatively small sample size (∼200 samples) and modest marker density (∼6,000 SNPs) in this study indicate caution against overinterpretation. Previous research has shown that at sample and marker sizes like ours SNP effects may be upwardly biased and can produce false positives (Slate, 2013). Furthermore, at such marker densities, heritability estimates are expected to be downwardly biased, although our estimates are consistent with studies on the same trait in closely related bighorn sheep (Coltman et al., 2003; Poissant et al., 2008; Miller et al., 2018). Ideally, these results should provide starting material for a validation study with finer scale genomic coverage using a separate population of Dall’s sheep and more individuals.

Previous studies of Soay sheep, a free-living feral breed of domestic sheep found on St. Klida archipelago (Scotland), have found a strong candidate for horn morphology in the gene *RXFP2*, located on chromosome 10 of the domestic sheep genome (Johnston et al., 2011). QTLs of suggestive significance on chromosome 10 have also been identified in bighorn sheep that are colocalized in the region mapped to *RXFP2* in domestic sheep (Poissant et al., 2011). Our post QC genotype data contained one locus (OAR10_29685536) in *RXFP2* gene, and this locus is not significantly associated with any horn size trait. In the 413,000 bp [found by Miller et al. (2018) to be the half-length of LD in bighorn sheep] region upstream and downstream of *RXFP2*, all 162 loci were monomorphic. However, more recent research based on whole-genome resequencing of six pooled bighorn sheep populations indicates a selective sweep of the *RXFP2* region consistent with positive selection (Kardos et al., 2015). Similar selective sweeps of *RXFP2* have also been found in domestic sheep (Kijas et al., 2012). So while it may have been somewhat unexpected to find no association of between *RXFP2* and horn size in thinhorn sheep, the presence of selective sweeps would prevent any signatures of association from being detected using our methods. We were unable to evaluate hypotheses regarding a selective sweep since a recent study has indicated that cross-species application of SNP chips is problematic for identifying runs of homozygosity, a typical signature of selective sweeps (Shafer et al., 2016).

While quantitative genetic and association studies have long been performed for some wild animals, most are part of large longitudinal studies. These types of projects can be costly and logistically challenging to operate, thus putting them out of the reach of most wildlife researchers. By taking advantage of the “genome-enabled” (Kohn et al., 2006) status of thinhorn sheep, we gained access to a high-density SNP array, which gave us a final SNP panel equivalent to that of a medium-density array (∼6,000 SNPs) with no cost toward marker discovery. Combining this SNP panel with recently developed methods for estimating genetic covariance using a genomic-related matrix allowed us to circumvent the need for a pedigree, which is available only in very few wild species at great cost and labor. Further, we utilized annuli produced by seasonal growth patterns as a form of repeated measure, thus giving us greater power than would have been otherwise available for the given sample size. Our approach leverages (1) cross-species application of domestic genomic resources, (2) existing governmental databases, and (3) annualized growth patterns to cost-effectively perform a first quantitative genetic and genome-wide association study for thinhorn sheep and one of the first for a wild species outside long-term active monitoring. While we caution against any overinterpretation of our results giving the limitations of our sample size and marker density, any of the three prongs in our analytical approach can be used by other studies to improve the cost-effectiveness, speed, and/or power of their analysis.

## Data Availability Statement

The data is available at EVA under the following accessions - Project: PRJEB34082; Analyses: ERZ1066075.

## Author Contributions

ZS conceived of the study. DC and ZS designed the study. ZS performed the data analysis and wrote the manuscript. DC provided input in results interpretation and provided substantial feedback on the manuscript.

## Conflict of Interest

The authors declare that the research was conducted in the absence of any commercial or financial relationships that could be construed as a potential conflict of interest.
