# Supplementary figures and images for: Heritability of Horn Size in Thinhorn Sheep
Source: Front Genet. 2019 Oct 11;10:959. doi: 10.3389/fgene.2019.00959 (PMC6797622; doi:10.3389/fgene.2019.00959)

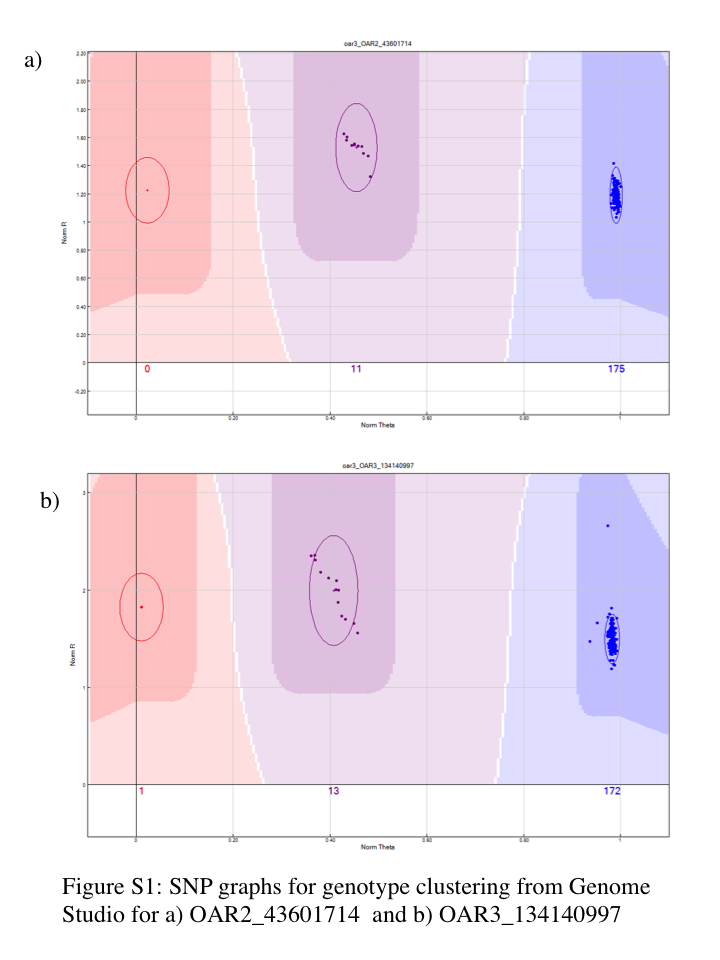

Supplement: Supplementary file 1 [file Image_1.png]
